# Medical Opinion and Movement

**Published:** 1910-11-12

**Authors:** 


### Medical Opinion and Movement.

### Hereditary Tuberculosis.

At a recent meeting of the Académie des Sciences Drs. Landouzy and Laederich gave an account of their researches into the transmission of tubercle bacilli from the maternal organism to the fœtus. A hundred and forty-three little guinea-pigs born of tuberculised mothers were examined. Fifty-seven of these, killed either *in utero* or immediately after birth, showed no evidence of tuberculous lesions either macroscopically or microscopically, nor bacilli in the viscera. On the other hand, the viscera of four fœtus taken from the uterus of a tuberculised guinea-pig and inoculated into two healthy guinea-pigs rendered them tuberculous. Sixty-eight guinea-pigs born of tuberculous mothers were allowed to live and were killed at the end of a few months, and of these sixteen showed definite tuberculous lesions localised exclusively in the lungs. These facts would appear to support the theory of Baumgarten that children born of tuberculous mothers may be infected with a latent tuberculosis which only develops at adolescence or even later. With regard to the transmission of a hereditary taint the authors note a high mortality among the offspring of these tuberculous animals and the frequent occurrence of congenital malformations, amounting to 4.6 per cent. Their observations would seem to confirm the view that the transplacental toxi-infection from a tuberculous mother tends to enfeeble the offspring and cause a degeneration of the race.

### A New Tuberculin.

Dr. F. J. Rosenbach, of Göttingen, has succeeded in preparing a new tuberculin by growing the fungus Trichophyton holosericum album on a culture of living tubercle bacilli. An account of his work appears in the *Deutsche Medizinische Wochenschrift*. The trichophyton grows luxuriantly on this culture medium, and the tuberculin resulting from this biochemical interaction is employed without being heated. The advantages claimed for the product are that there is no intereference with the immune substances by heating, and that the poisonous by-products of the growth of the tubercle bacilli are destroyed by the action of the trichophyton. The tuberculin is employed for its local as well as for its general effects. From observations on animals there is leucocytic infiltration of the tuberculous tissue following the injections, accompanied by exudate formation and later absorption. The tuberculous focus thus degenerates and then heals. If there is already caseation, suppuration may follow; but if there is outlet for the pus, healing may result. In cases of surgical tuberculosis the tuberculin is injected locally into the lesion. The reaction is then much more intense. Even acute phlegmonous areas may appear, but they subside spontaneously and are followed by healing. Rosenbach refrains from declaring that his tuberculin is a curative agent, but he states that the immediate results are excellent, far better than the results with other forms of tuberculin.

### Appendicostomy for Chronic Dysentery.

It is now some time since appendicostomy or cæcostomy was advocated in the treatment of chronic dysentery, chronic colitis, and allied conditions, in order to allow of thorough and repeated irrigation of the lower bowel. A report on thirty-one cases is published by Dr. Walter D. Webb in which the operation has been carried out. The patients suffered from chonic amœbic dysentery or chronic colitis following acute dysentery. The operation of appendicostomy followed by irrigation gives satisfactory improvement in the symptoms, but does not suffice to produce cures. If the openings are allowed to close, the dysentery returns. In the amœbic cases the parasites rapidly disappear during the treatment. In five cases cæcostomy was performed; but the author is of opinion that appendicostomy is to be preferred, and should always be chosen unless there is some special reason against its performance. There appears to be a great tendency to weakening of the abdominal wall, and in eight cases a ventral hernia took place.

### A Case of Leprosy by Inoculation.

Dr. W. Russell publishes, in the *South African Medical Record*, the interesting case of a boy of fourteen, son of a Dutch farmer, who contracted leprosy by direct inoculation. No history of the disease could be traced in the family. The boy cut his foot on a piece of broken bottle while bathing, the wound bleeding freely. A native aged twenty passing at the moment suggested putting tobacco on the wound to stop the hæmorrhage, at the same time producing from his mouth a piece he had been chewing. This was applied to the cut, which was bound with a handkerchief and a boot put on. Returning to school, the wound kept breaking out every two or three months, with evacuation of pus. This continued for about three years, when the medical man consulted, suspecting bone necrosis, cut down and scraped, the wound subsequently healing slowly. A rash then developed on the body and the boy became ill. In a few months his nose and throat commenced to give trouble, and he became hoarse. Syphilis was diagnosed by several consultants, and antispecific remedies tried for nine months but without avail. He then came under the author's observation. He was fairly well-nourished, talked in a husky voice, had no ulceration but much congestion of the mucous membrane of the throat. There was slight nasal discharge. His hair was brittle and falling out, no rash was to be seen on his body, and the original scar on his foot was healed. Diagnosis was then syphilis and treatment instituted. In six weeks no improvement occurred, the hoarseness increased, the shooting pains in the limbs were no better, and the temperature rose two or three degrees from time to time. Various consultations were held and the diagnosis confirmed by other observers. He got progressively worse under treatment. The nasal secretion was then examined for the first time, and typical *Lepra bacilli* found. The author had the boy under observation for six months, during which typical leprous manifestations developed gradually, such as the characteristic expression, eyebrows denuded of hair, anæsthetic patches on the body, ulcers on the right foot and right vocal cord, and painful fissures between the toes. He lived about eleven years from the original inoculation. Inquiries made regarding the native who had supplied the tobacco showed that he was a leper coming from a leprous family. He died four years after the presentation of his fatal gift.

### Hysterical Aural Hæmorrhage.

A VERY unusual case is reported by Dr. Kutvirt in the *Revue Neurologicka* of hæmorrhage from the ears, believed to be of hysterical origin. He has found half a dozen similar cases recorded. His patient was a girl about fourteen years of age, who began to suffer from spontaneous hæmorrhages from the left ear. At first these occurred at intervals of a few weeks, but not in any cycle suggesting a menstrual connection; the amount of blood lost was about half an ounce, and both ears were quite normal to inspection. As time went on the hæmorrhage became steadily more frequent, and at the end of a year the right ear also became similarly affected. The attacks were provoked only by excitement; the patient was extremely irritable, and would begin to cry for very little cause. Eventually the bleeding attacks came on three or four times a day. There was no diminution of auditory acuity, nor any abnormality of the ear, except hyperæsthesia of the concha, auditory meatus, and tympanum. The visual fields were both normal, and there was marked tenderness of both ovaries. Exactly what the explanation of this extraordinary phenomenon was is very difficult to judge; but it certainly seems as if the diagnosis of hysteria is at least a possible one, though the case still remains distinctly mysterious.

### The Action of Tyramine.

THE physiological actions and therapeutic applications of tyramine have been investigated by Dr. A. Clark, who publishes his results in the *Bio-Chemical Journal*. Tyramine, also known as ergotamine, is an organic base which can be produced from tyrosine by the action of certain bacteria normally existing in the alimentary canal. It is found in placental extracts, probably formed by incipient putrefaction, and is the principle mainly responsible for the pressor effects of such extracts when injected. Dale and Dixon have thoroughly investigated the effects of tyramine (parahydroxy phenylethylamine) upon animals, and find it produces a marked rise of blood-pressure, partly by augmentation of the cardiac output and partly by vaso-constriction. Its action is similar to that of adrenalin, but is slower and more sustained; and the local action is less. A more important difference is that it can be given either hypodermically or by the alimentary canal. The conclusions summarised by Dr. Clark are: (1) Tyramine, when given by the mouth to a healthy subject, in doses of 30 to 100 milligrammes, causes a slight rise of blood-pressure, which lasts for some hours. (2) When injected hypodermically into a healthy subject in doses of 20 to 50 milligrammes, it causes a rapid and well-marked rise of blood-pressure, lasting for about twenty minutes. (3) When injected hypodermically in doses of 20 to 50 milligrammes into patients in a condition of shock, tyramine causes a slight rise of blood-pressure. This effect is seen even in cases of extreme collapse. It seems as if further clinical experience will be quite worth having as to the value of this substance for the treatment of shock.

### Telephone Neurosis.

As the result of his researches into the maladies to which female telephone operators are subject, Dr. Thébault states in *La Presse Médicale* that they are the effect of stress of work on the predisposed individual, and not of the use of the instrument itself. The use of the telephone leads to no disease which can be called occupational in the sense of those which follow in the train of laundry-work, the stonemason's trade, etc. The series of facts which he reports prove, nevertheless, that a certain number of these young women contract disorders which result from their occupation, as is shown by the disappearance of symptoms on their seeking rest or on their becoming used to their work. Disorders complained of are almost entirely nervous, and vary in kind in different individuals. In some they may be traced to irregularities of diet increased by overwork. Migraine and tinnitus lasting for days at a time may be a source of trouble until the patient becomes inured to them. In others, depression, melancholia, misanthropia, hysterical gaiety, irritability, loquacity, a tendency to weep, or even sullen indifference are noted. The author's findings agree with those of Dr. Blegvad, who investigated disorders among the female telephone operators of Copenhagen.

### Dermatitis from Balsam of Peru.

A CASE of extensive skin trouble resulting from the use of Peruvian balsam for the cure of scabies has been reported by Hazen in the *Journal of the American Medical Society*. When the patient had applied an ointment of the balsam freely to the hands and arms, the whole body broke out into an ill-defined macular eruption. The use of a mild lotion and discontinuance of the ointment led to the speedy disappearance of the rash. The majority of cases derive nothing but benefit from the balsam of peru treatment of itch, but in addition to the macular eruption mentioned above some patients have developed urticaria; others have attributed bronchitis to the drug; and nephritis has developed in a few instances.

### Medical Effects of Late Marriage.

In a *Thèse de Paris* Dr. Le Hir publishes the conclusions at which he has arrived as the result of his investigations into 724 marriages among elderly people. He has adopted as a definition of what constitutes a late marriage one in which the age of the man is fifty or over, and that of the woman thirty-five or more. If the vitality of a child be measured by the rate at which it resumes the weight it possessed at birth, there is an undoubted enfeeblement among children born of elderly parents. The majority of new-born infants resume their original weight between the fifth and seventh day following that of birth. In cases where the parents are middle-aged this resumption is delayed to between the ninth and eleventh day. Again, if pregnancy has been complicated by albuminuria not sufficiently advanced to cause the death or premature expulsion of the fœtus, the effect of the disorder will be reflected both on the actual weight of the child and on the rate of resumption of this weight. However, pregnancy will advance on physiological lines, resulting in an easy delivery and a normal child in spite of the parents being elderly so long as they are not in a period of marked general retrogression and are free from taint and in good health, and so long as the mother has been cared for in every respect. On the other hand, maladies occurring in middle-aged parents will have evil effects on the offspring if digestive and functional derangements have brought about a disintegration of the constituent elements of the cells, and consequently a general denutrition and organic decay.

### Multiple Vesical Calculi.

Dr. Raubenheimer publishes in the *South African Medical Record* an account of a case from which an unusually large number of vesical calculi were removed. The patient, a carpenter, aged sixty-three, and weighing some fourteen stone, came under observation complaining of frequency of micturition, a sensation of weight in his bladder, severe pain at the point of the penis, and scalding after micturition. His temperature was 103°, his pulse 115, and he had frequent rigors. He had passed urine thirty time in eight hours during the previous night. On passing a catheter the author drew off about half an ounce of foul-smelling urine containing pus and phosphates. No difficulty was experienced in passing the catheter, but the presence of foreign bodies in the bladder could be distinctly made out even with the soft catheter used. With a view to getting rid of the superadded infection the bladder was washed out daily for six weeks, after which the temperature became normal, though the frequency of micturition never fell below fourteen times in eight hours, and the pain and weight remained the same. The bladder was then opened through a supra-pubic incision in the middle line, and seventy-three phosphatic calculi removed. These together weighed half a pound, the larger ones being the size of a child's marble. Two of them were partially encysted at the fundus of the bladder. The wound was left open, a drain inserted, and the bladder washed out in the usual way. Recovery was uneventful, the patient being able to get up on the twenty-fourth day. He has since been in good health and able to follow his trade.

### Sloughing of the Cornea.

Keratomalacia, or gangrene of the cornea, is a complaint which Mr. Sydney Stephenson deals with exhaustively in a contribution to the *Ophthalmoscope*. It is found in practically every country on the globe, and affects especially children from three to twenty months of age; it is more frequent about the eighth month of life. In half of the cases both eyes are affected, and it is chiefly the infants of the poorest classes that suffer. Half of the cases are associated with xerosis conjunctivæ. Keratomalacia is found only in children whose vitality has been seriously reduced by epidemic enteritis, congenital syphilis, athrepsia, or tuberculosis, named in their order of frequency. In London its seasonal incidence follows closely that of zymotic enteritis. The mortality amounts to about 50 per cent. of those affected; the immediate cause of death is usually broncho-pneumonia or exhaustion. Of those who survive about half remain blind. No specific micro-organism has been proved to be the infecting agent, but scrapings from the corneæ of affected babies yield the pneumococcus in about one-half of the cases. Besides general treatment, and the local use of fomentations and antiseptic washes, the author places faith in physostigmine and pilocarpine in oily solution.

### Fistula of Colon from Intestinal Worms.

Dr. Stocker de la Rosa publishes in the *Rivista de Medecina y Cirugia Practicas* a remarkable case of fistula of the colon due to intestinal worms. A child of seven had suffered a few months before examination by the author from severe intestinal obstruction accompanied by rigors, fever, and fixed pain localised to the right region of Petit. This attack was followed by local inflammatory symptoms, after which an abscess developed in the above-mentioned locality; this burst spontaneously and gave issue to worms and fæcal matter. A fæcal fistula remained after this which from time to time healed, only to break down again after a period of cure. While the fistula remained closed fæcal matter was passed *per vias naturales*. An operation was advised and agreed to. A large mass of cicatricial tissue was resected. On arriving at the bowel the different layers were sewn up to their corresponding ones, and the peritoneum from neighbouring healthy parts tied over the bowel *en masse*. After returning the parts into the abdomen, the wound was closed in the usual way, adequate drainage being provided. In a few days a complete cure resulted. During the operation the author was able to discover the cause of the sinus becoming occluded from time to time. A flap of intestinal mucous membrane obtruding through the rigid cicatricial ring of the fistula acted as a cork on occasions, completely occluding the passage.

